# Evaluation of a Method for Assessing Food Contamination Based on a Shopping Mall Model

**DOI:** 10.3390/foods14173110

**Published:** 2025-09-05

**Authors:** Marcin Niemcewicz, Rafał Szelenberger, Weronika Grabowska, Natalia Cichon, Marcin Podogrocki, Michal Bijak

**Affiliations:** Biohazard Prevention Centre, Faculty of Biology and Environmental Protection, University of Lodz, Pomorska 141/143, 90-236 Lodz, Poland; marcin.niemcewicz@biol.uni.lodz.pl (M.N.); rafal.szelenberger@biol.uni.lodz.pl (R.S.); weronika.grabowska@biol.uni.lodz.pl (W.G.); natalia.cichon@biol.uni.lodz.pl (N.C.); marcin.podogrocki@biol.uni.lodz.pl (M.P.)

**Keywords:** food defense, CBRN, risk factors

## Abstract

This study evaluated a novel methodology for assessing food safety vulnerabilities in shopping malls by integrating Hazard Analysis and Critical Control Points (HACCP), Threat Assessment and Critical Points (TACCP), and Failure Mode and Effects Analysis (FMEA). Inspections were conducted in nine shopping centers across Poland, the Czech Republic, Slovakia, and Spain to identify the risk of intentional/unintentional contamination with chemical, biological, radiological, and nuclear agents. The assessment considered key operational areas, including food delivery, transportation, staff security, back-office access, product handling, and inspection protocols. Risk levels were quantified using FMEA parameters. The findings revealed an overall high to average risk score with the most critical vulnerabilities linked to back-office access, unauthorized personnel entry, and susceptibility to fraudulent inspections. Observations also highlighted infrastructural shortcomings, insufficient monitoring, and procedural gaps that could facilitate contamination. The proposed methodology offers a structured, quantitative framework for identifying and prioritizing food safety hazards in public environments. Implementing targeted countermeasures—such as enhanced surveillance, strict access control, staff training, and dedicated food handling protocols—can substantially reduce risks, thereby strengthening public health protection and operational resilience. This approach may serve as a promising framework for integrating food defense and safety assessments for food defense in high-density commercial facilities.

## 1. Introduction

Protecting the food supply chain from deliberate contamination or accidental exposure to chemical, biological, radiological, and nuclear (CBRN) agents is one of the key challenges for modern security systems. In an area of global trade and complex distribution networks, ensuring food safety requires not only traditional quality control measures but also a comprehensive approach to potential CBRN threats [1]. Food distribution centers, particularly shopping malls with extensive food courts and supermarkets, serve as critical nodes in urban food security infrastructure. These locations are especially vulnerable, as the release of CBRN agents could affect a significant portion of the population, making them essential elements of the overall food protection strategy [2]. Research at the intersection of food security and CBRN threats contains several key functions. It enables early detection of emerging threats and helps identify weaknesses in the food security system. It also supports the development of improved food safety methodologies, effective decontamination protocols, and resilient distribution networks. Furthermore, this research informs the creation of regulatory frameworks, operating protocols and practical guidelines for the food service and retail sector.

Ensuring food safety and security is a critical aspect of modern food production and distribution. To mitigate risks associated with food contamination, several analytical methodologies are employed, including Hazard Analysis and Critical Control Points (HACCP), Threat Assessment and Critical Control Points (TACCP), and Failure Mode and Effects Analysis (FMEA).

HACCP is a systematic, preventive approach used to identify, evaluate, and control biological, chemical, and physical hazards in food production. It focuses on critical control points (CCPs) to ensure food safety throughout the supply chain [3,4]. In contrast, TACCP is a risk management system designed to address intentional threats, such as food fraud, sabotage, and bioterrorism, integrating vulnerability assessments to protect food products from malicious contamination. Meanwhile, FMEA is a structured methodology used to identify potential failure modes within processes and assess their impact on product quality and safety. By prioritizing risks based on severity, occurrence, and detection (also referred to as vulnerability, impact, likelihood), FMEA enhances proactive decision-making in food production [5]. While HACCP, TACCP, and FMEA are well-established individually, their combined application has not yet been operationalized into a single harmonized framework for food defense. Our approach fills this gap by: introducing quantitative severity-likelihood-detectability scoring into TACCP. Furthermore, aligning hazard and threat assessments on a common risk scale, and creating a unified tool for both unintentional and intentional food risks. This differs from existing frameworks, which tend to address these risks in parallel rather than in an integrated, system-wide manner.

In the context of shopping centers, critical points often emerge at intersections of different operational domains. For instance, the handling of food waste across multiple food service establishments represents a potential critical point where biological hazards could proliferate if not properly managed.

That is why we developed an innovative approach to address this research gap, specifically tailored for food safety assessment in public spaces, particularly shopping centers. To date, the most prominent documented case was in 2003, a Chi-Chi’s restaurant located in the Beaver Valley Mall, Monaca, Pennsylvania, experienced a Hepatitis A outbreak, traced to contaminated green onions. The outbreak affected 527 people, with 485 hospitalization status and 3 deaths [6]. Furthermore, Dingsdag et al. performed a study in which tables and cleaning cloths in food courts at shopping malls were investigated to highlight potential risk of microbial contamination. Results showed high levels of bacteria, including opportunistic pathogens like *Stenotrophomonas maltophilia* and *Eromonas hydrophila* on cleaning cloths and table surfaces, suggesting a risk of a cross-contamination [7].

The evolving nature of CBRN threats necessitates continuous adaptation of existing food safety protocols, especially in high-density commercial environments such as shopping malls, where the convergence of food preparation, storage, and consumption creates complex security challenges. These challenges require interdisciplinary approaches that integrate expertise from food science, security studies, public health, and crisis management. Mall operators bear significant responsibility within this ecosystem, serving not only as commercial owners but also as managers of critical infrastructure. They must implement comprehensive food defense strategies that address both overt attack scenarios and more subtle contamination methodologies. This requires close collaboration with tenants, regulatory bodies, and security services to establish unified response capabilities and consistent safety standards. For all of these reasons, we conducted a research analysis focused on establishing the food safety and security in shopping malls.

## 2. Materials and Methods

### 2.1. Construction of the Study

The data for this study were collected during the research phase of the Mall-CBRN project, funded by the European Union’s Internal Security Fund—Police Grant Agreement No. 861643 [8]. The project consortium consisted of civil and military research institutions, CBRNe protection specialists, and representatives from shopping centers belonging to major international retail chains.

The primary objective of the project was to develop and implement a comprehensive CBRNe protection system tailored to the specific needs of large shopping centers. Food defense is a fundamental component of this system, encompassing an awareness-raising and training program, along with two key reports: Best Practices for the Prevention of Food-Related CBRN Incidents and Countermeasure Procedures in the Event of Food-Related CBRN Events. All those developments were based on the findings from nine inspection visits conducted at shopping centers in Poland, the Czech Republic, Slovakia, and Spain. As these centers form part of the international retail chains, the research may be considered broadly indicative of the food safety status in EU countries.

**Figure 1:**
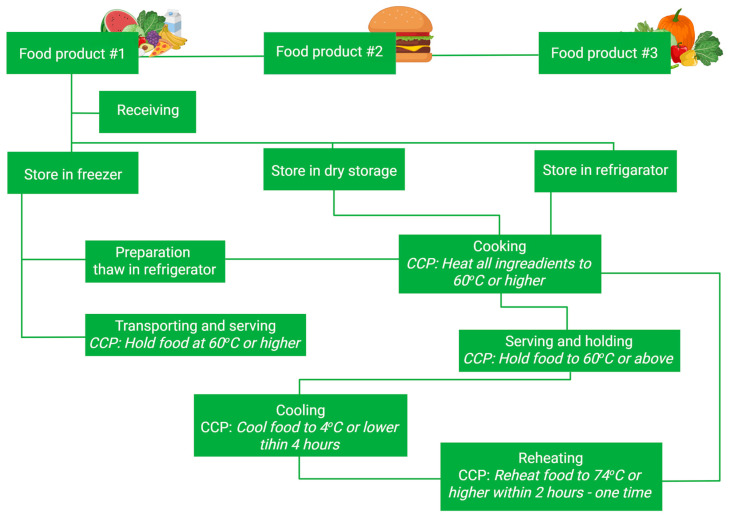
A flow diagram illustrating the food product processing within the HAACP guidelines and showing all the critical control points. Created in BioRender. Bijak, M. (2025) https://BioRender.com/d8li2rb (accessed on 28 August 2025).

**Figure 2:**
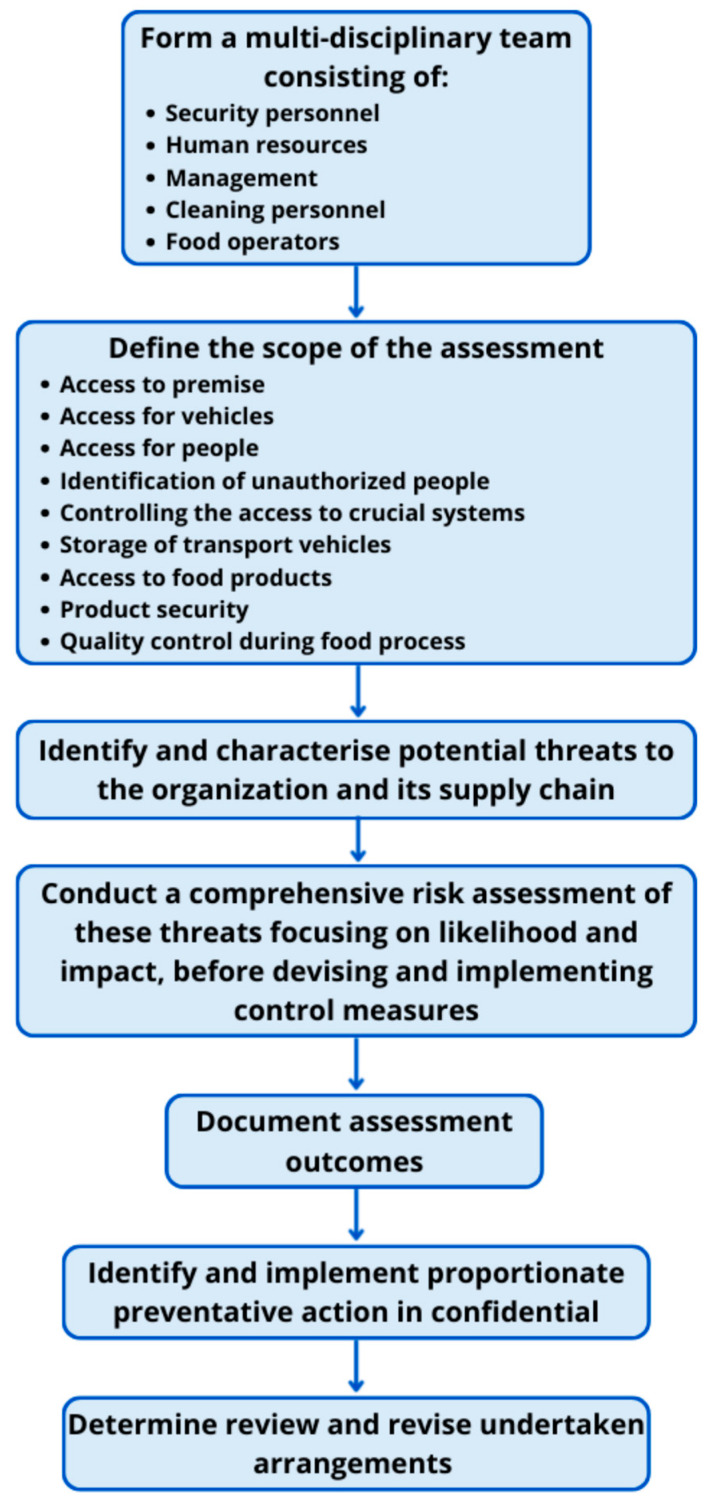
A flow diagram illustrating the routes for TACCP.

For the purpose of a comprehensive analysis, the integration of Hazard Analysis and Critical Control Points (HACCP), Threat Assessment and Critical Control Points (TACCP), and Failure Modes and Effects Analysis (FMEA) was carried out in a balanced manner. HACCP and TACCP were used to define relevant hazards and threats, while FMEA was applied to prioritize them within a unified risk management framework. A flow diagram illustrating HACCP and TACCP routes is presented as Figure 1 and Figure 2.

### 2.2. Methodology of FMEA

In this method, quantitative risk assessment is derived using an equation that integrates three parameters: vulnerability to threat (V), the potential impact of the threat (W), and the probabilistic likelihood of its occurrence (PR). The mathematical representation of this risk calculation is as follows:

R=V×W×PR

Each component of this risk assessment is evaluated using a five-point scale, providing a structured approach to quantifying potential risks and their associated characteristics.

The parameters vulnerability (V), impact of the threat (W), and likelihood of a hazard occurrence (PR) were selected in order to adapt the classical FMEA scoring system to the specific requirements of food defense, and were presented in Table 1, Table 2 and Table 3. While traditional FMEA relies on Severity, Occurrence, and Detectability, the latter is less meaningful in the context of intentional adulteration, where detection may occur only after consumption. Instead, impact of the threat (W) reflects the strength of preventive and mitigation measures, consistent with PAS 96 [9] and the FDA’s Food Defense Plan Builder [10], both of which emphasize the role of control robustness. vulnerability (V) captures the product, process, or supply chain node to attack susceptibility, aligning with approaches recommended in TACCP and food fraud vulnerability assessments [11,12]. Finally, likelihood of a hazard occurrence (PR) integrates the likelihood of exploitation with the system’s ability to withstand or recover from disruption, following broader risk management principles [13]. This adaptation ensures that the scoring system reflects both feasibility of attack and organizational resilience, providing more realistic prioritization of food defense risks.

**Figure 3:**
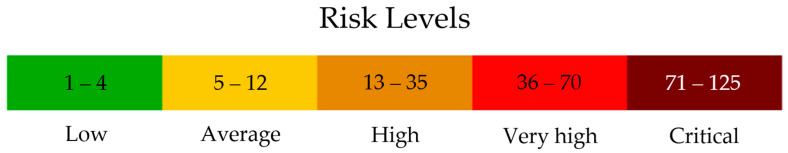
Gradient bar presenting adopted risk scale.

Based on the results obtained and the adopted scheme, the product of the tested parameters determines the risk level for individual points of the food safety chain. The adopted risk scale (product) is presented in Figure 3.

To estimate the risk related to food safety and security within an organization, the following CCPs were selected for investigation (Table 4). These critical control points were identified through a review of the literature [14,15,16,17,18], consultation with management staff, and fact-finding missions conducted in various shopping centers during the initial research phase of the Mall-CBRN project. Inspection visits were conducted according to a standardized protocol and structured templates, developed in collaboration with shopping mall management to reflect specific operational conditions and ensure uniformity in data collection and analysis. Each inspection lasted a full working day (approximately 12 h), encompassing the entire food service cycle, from delivery and preparation through service to post-closing cleaning. The protocol included a semi-structured interview with representatives of management, technical, and security staff, followed by a targeted on-site inspection of selected areas. All visits were carried out by a multidisciplinary team of four evaluators, comprising one professor, three PhD holders (with one Doctor of Veterinary Medicine) with expertise covering sanitary inspection, food safety, microbiology, biochemistry, epidemiology, hygiene, and public space security, which ensured methodological rigor, consistency, and reliability of the collected observations. Each expert received the same checklist and independently assessed the parameters according to their judgment. The completed checklists were then collected, and the scores for each parameter were averaged across all experts.

## 3. Results

**Figure 4:**
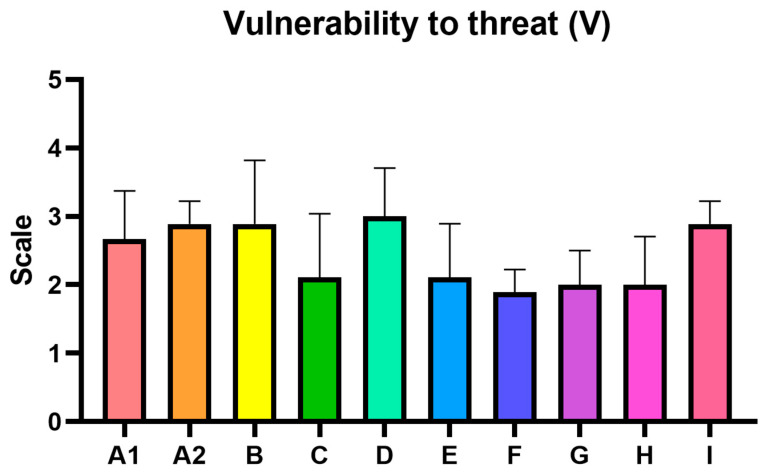
Vulnerability to threat (V). Each bar present mean ± standard deviation for the critical control points.

**Figure 5:**
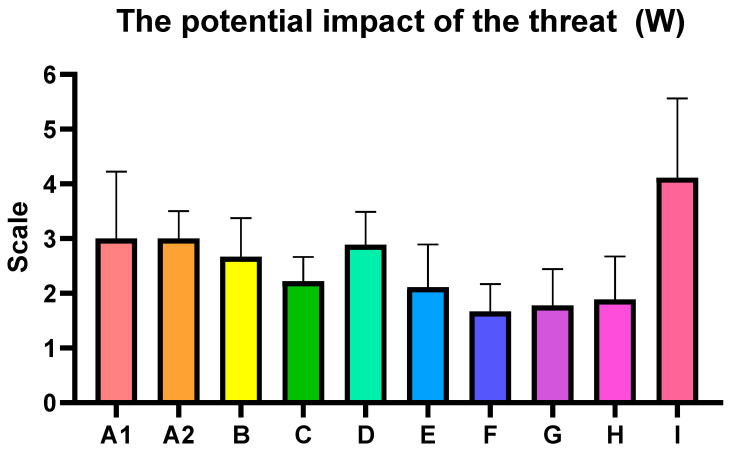
The potential impact of the threat (W). Each bar present mean ± standard deviation for the critical control points.

**Figure 6:**
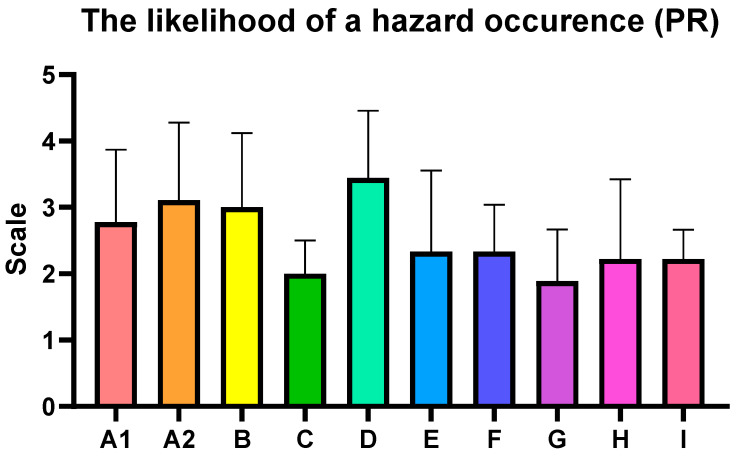
The likelihood of a hazard occurrence (PR). Each bar present mean ± standard deviation for the critical control points.

The following assessments summarize the key findings from the analysis, highlighting the identified food safety risks and CBRN hazard vulnerabilities observed during the inspection visits to nine diverse shopping centers in Poland, the Czech Republic, Spain, and Slovakia. The average risk level for the inspected centers was estimated at 22.22, which falls within HIGH category (13–35), indicating the need for corrective interventions (Table 5, Figure 4, Figure 5 and Figure 6). What is noteworthy, there were no specific differences between studied countries.

### 3.1. Food Delivery—Access to the Shopping Center (A)

Food product deliveries take place either through the back office or, occasionally, through the main entrance by couriers (although this practice is not common). The back-office area is generally equipped with monitoring system and in most inspected facilities, secured by a barrier. However, the monitoring system in the supply area is typically insufficient. The delivery ramp is used for all types of products. No ramp is dedicated solely to food deliveries, even where multiple ramps (e.g., four) exist. In shopping centers, there is usually one ramp used for all delivery. According to the information obtained, no specific times were designated exclusively for food delivery, and food shipments are often carried out together with other goods. Dedicated supervision of food supplies was not observed. In most cases, the routes for garbage disposal and food transportation were the same and often intersect. Some facilities also allowed direct access to shops and restaurants directly from street parking areas. The buildings are designed so the that entrances face open common areas, with each store having its own separate doors and escape routes, most often through its main entrance. Catering establishments follow a similar pattern. Rather than forming a single food court zone, they consist of individual restaurants, each with its own kitchen facilities and supply area. Every restaurant has a separate internal dining area, and some extend this with fenced outdoor dining areas to the restaurant. The food supply chain is managed by the individual restaurants, with the shopping center’s security notified of deliveries in advance. No catering points with a self-service options were identified, which reduces the risk of food contamination by unauthorized personnel.

Access for individuals through back-office facility is monitored in a manner similar to freight deliveries. However, the existing monitoring system is, in most cases, ineffective, creating a high likelihood that outsiders could pass through unnoticed. Although, the doors are equipped with standard locks, inspections revealed instances where they had been left open. Furthermore, the large number of suppliers reduces attentiveness to the presence of unauthorized individuals.

### 3.2. Food Transportation Inside the Facility (B)

In most of shopping centers visited, a lack of specific transport for food products was observed. There were no designated ramps leading to back office, and transportation to higher floors was carried out using the same elevators as other supplies (e.g., chemicals and products intended for distribution to retail stores). In most cases, the lifts were connected directly to the delivery ramp. Despite the availability of multiple elevators, none were reserved exclusively for food products transportation. It was observed that the same elevators were also used for waste transport. Additionally, no dedicated delivery hours for food products (including fresh vegetables and fruit) were established in the inspected centers. Food deliveries were often left unattended in front of closed restaurants by couriers who dropped off their products. Due to the layout of the shopping center, in some cases products were transported only through designated supply points within the restaurant.

### 3.3. Security Staff (C)

Security staff are responsible for monitoring certain aspects of the food safety system that fall under the owner’s scope of supervision. Since catering establishments are, in most cases, rented premises, oversight from the ownership perspective is limited. However, it should be noted that the overall level of risk awareness among security staff is appropriate. They document observed irregularities and reporting them to their superiors. According to information gathered during inspection visits, the training process for security guards also includes food safety aspects. Inspections revealed well-developed monitoring systems, supported by the strong involvement of security staff. Nevertheless, at several sites, the awareness of risks related to food chain safety among security personnel was found to be insufficient or only moderate.

### 3.4. Back-Office Access (D)

In most inspected shopping malls, access to the back-office catering facilities both within and outside the food court was controlled by electronic cards, which allowed doors to be opened or locked. However, during the on-site visits, it was frequently observed that these doors had been left open, permitting access by outsiders. According to the information obtained, the primary cause of this issue was the frequent loss of access cards and/or keys by catering facilities employees. This situation poses a serious threat as it enables unauthorized individuals to gain access unnoticed and without employees intervention, potentially allowing acts of sabotage aimed at contaminating food products. Inspections also revealed that most shopping centers had an insufficient number of cameras monitoring catering facilities. For example, during one inspection, only a single camera was positioned at the entrance door, covering only a small section of the back office. In addition, inadequate oversight of cleaning companies was noted. In several cases, storage room for household chemicals was left open and unattended. In the back-office corridor of catering facilities, cleaning residues and household chemicals were sometimes stored in close proximity to freshly delivered food products, such as fruit. Overall, direct access to the back of catering facilities both inside and outside the food court was observed during on-site visits. Doors were often left open, allowing unrestricted entry to passers-by. This condition presents a significant security risk, as unauthorized individuals could enter unnoticed and potentially engage in sabotage aimed at contaminating food products.

### 3.5. Ready-Made Gastronomic Products Sold in an Open Manner (Following Free Access to the Product by Third Parties) (E)

During a simulation conducted to assess the potential for contamination of finished and displayed food products, it was found that in some facilities, act of sabotage such as food contamination by third parties before delivery to the customer could be carried out without any reaction from employees. In addition, a small number of facilities lacked physical security measures to prevent such incidents. However, in most locations, it was demonstrated that sabotage through food contamination before delivery was not possible, as the food was properly protected from external access. On-site visits confirmed that food products were generally well secured, while also identifying areas for improvement in food safety. In several establishments, the handling of finished food products was closely supervised from the moment of ordering until delivery. In these cases, food was stored behind glass barriers and positioned at a distance, making sabotage attempts significantly more difficult. Food security measures varied across establishments. In 40% of the premises, food was protected using plastic or glass barriers, while in the remaining facilities, direct access to prepared food was possible. Overall, inspections confirmed that food products were typically well protected. Displayed food was either secured behind glass or both shielded by glass and placed away from customers, further reducing the risk of contamination. In some shopping centers, food outlets operate as independent zones with their own kitchen facilities and supply areas. Each catering point has a separate internal dining area, and no self-service options were found. This setup effectively eliminates the possibility of food contamination by unauthorized personnel.

### 3.6. Preparation of the Consumption Areas to Serve Guests Eating Meals on the Spot (F)

In the visited shopping centers, the dining areas were prepared in accordance with the applicable sanitary guidelines (e.g., hand sanitizers, disposable gloves, information boards). In most facilities, a security guard was present, continuously monitoring the dining area. Access to food products was secured, and food display cases were covered and protected. It should be emphasized that the space were properly prepared for both on-site dining and takeout. However, in most facilities, the number of surveillance cameras was insufficient, which could hinder effective supervision of the food court area.

### 3.7. Preparation of Food Products for Distribution (G)

The lessee of the premises cannot interfere with the meal preparation process carried out by the tenant and/or their employees. However, it is recommended that security personnel monitor the behavior of food and beverage staff, for example, via a surveillance system. Any atypical or suspicious behavior, such as frequent absence from the workstation, bringing in unauthorized items, sudden changes in behavior, or signs of nervousness, should be observed and recorded. In such cases, it is advisable to promptly contact the tenant for verification. No significant shortcomings were identified during the on-site visits.

### 3.8. Ready-Made Products Service (Both Eaten on Site and Take-Away) (H)

To collect their ordered meals, which are typically consumed on-site in most areas of the shopping center food courts, customers wait for their turn. Orders are called out using an assigned number, order type, or electronic device. It was observed that, after meal preparation and customer notification, employees sometimes leave completed orders on the counter in an open and unsecured manner. These orders remain unattended until the customer arrives, with no staff supervision. The practice of prepared food dispensing without supervision or protection influences the overall risk assessment of this aspect of food service. Furthermore, the commitment and awareness of restaurant staff play a critical role in maintaining food safety. Self-service is not permitted.

### 3.9. The Probability of Inspection Carried out by an Individual Posing as a Public Health Inspector (I)

Based on interviews with employees responsible for safety at the shopping centers and a simulation conducted with female staff members posing as representatives of the corporate headquarters, it can be assumed that, in the event of a fraudulent food safety inspection, a similar procedure would like to occur. Specifically, individuals claiming to be representatives of the Public Health Inspectorate could gain the access to key areas of the shopping centers that are critical for food safety. A crucial factor in this scenario is the response of the center’s employees, who openly acknowledged the absence of established procedures for such situations, as well as the lack of verification protocols for individuals claiming to be authorized inspectors. These employees have expressed their willingness to address this issue promptly.

## 4. Discussion

The assessment of shopping centers regarding potential CBRN attacks targeting the food chain reveals several critical vulnerability areas that warrant attention. These findings align with previous food defense analyses that have highlighted infrastructural design, centralized catering arrangements, and human factors as recurring areas of concern in mass catering environments [11,19]. Infrastructure-related vulnerabilities in malls, including driveways, ramps, passageways, and elevator systems parallel those identified in airport catering and hospital food services, where access points can unintentionally double as contamination vectors [20,21]. The concentration of food production and distribution within centralized “food courts” creates efficiency benefits but also replicates the “single-point-of-failure” risks noted in study concerning food safety incidents [22]. In addition, scattered food outlets and delivery systems echo vulnerabilities in fragmented supply chains, where multiple contamination points require rigorous oversight. Human factors constitute a significant vulnerability, consistent with literature underscoring insider threats as a primary concern for food defense [9]. Rigorous pre-employee screening, comprehensive training programs, have been identified as best countermeasure practices across sectors, yet shopping centers face particular challenges due to high staff turnover in retail food service. Our findings reinforce these concerns and suggest that even moderate improvements in personnel management could substantially reduce vulnerability.

The feasibility of the proposed countermeasures varies considerably across shopping mall contexts. Measures such as stricter access control through ID badges, visitor logs, and staff training represent low-cost, high-feasibility interventions that can be readily integrated into existing security operations. Enhanced surveillance requires greater capital investment, but many malls already possess extensive CCTV networks that can be extended to cover food storage and preparation areas at relatively modest cost. By contrast, advanced biometric access systems or AI-enabled monitoring may be less feasible for most operators due to financial and logistical constraints, and could face challenges in staff compliance and acceptance, especially given high turnover in food service roles. Therefore, a phased implementation approach, prioritizing low-cost and high-impact measures, may offer the most practical pathway for shopping mall operators.

Overall, the observed vulnerabilities correspond to a medium-to-high risk profile for CBRN-related food chain disruptions in shopping malls, a result that is broadly in line with previous assessments of soft targets in public service settings [23]. Furthermore, the observed vulnerabilities in shopping centers also align with the expectations and guidance set forth by key food safety and defense regulatory frameworks. The FDA’s Intentional Adulteration (IA), rule emphasizes the identification of actionable process steps and points of vulnerability in food operations, particularly in public-facing or high-volume settings [24]. Similarly, PAS 96 highlights the importance of assessing both insider threats and infrastructural weaknesses in mass catering environments [9]. At the international level, Codex Alimentarius principles [25] and ISO 22000:2018 [26] call for systematic, risk-based management of food safety hazards, including those arising from intentional contamination, with continuous monitoring and verification. By operationalizing these principles through the integrated HACCP-TACCP-FMEA framework, this study provides a practical approach that complements existing regulatory expectations. It demonstrates how targeted countermeasures from enhanced access control to staff training and surveillance can be prioritized and implemented in accordance with both national and international guidance, thereby enhancing compliance, public health protection, and operational resilience in complex commercial food environments.

The challenges to food safety are multifaceted, encompassing biological contaminations that can originate anywhere from agricultural fields to serving points, as well as chemical contamination from environmental pollutants, agricultural residues, or inadequate sanitary conditions. The increasing accessibility of CBRN agents has elevated the risk of food products being used as contamination vectors. Nevertheless, these challenges can be effectively managed by implementing precautionary measures throughout the production, handling, transportation, and service stages of the food supply chain. Food remains an essential yet vulnerable resource that requires vigilant protection and forward-thinking security approaches. The proposed countermeasures will significantly reduce the risk of both intentional and unintentional food contamination, thereby enhancing public health protection while ensuring business continuity.

The integration of HACCP, TACCP, and FMEA provides a comprehensive framework for risk assessment in complex environments such as shopping centers. This methodology is based on several key assumptions that must be acknowledged before implementation. First, it assumes that all potential hazards can be identified through systematic analysis. Second, it presumes that the likelihood and severity of these hazards can be quantified with reasonable accuracy. Third, it operates under the premise that appropriate control measures can be established to mitigate identified risks. Finally, it assumes that continuous monitoring and verification processes can be effectively implemented.

The selection of critical control points within the HACCP framework traditionally relies on decision trees and expert judgment. However, this approach may lack the quantitative rigor necessary for complex environments like shopping centers. The FMEA methodology enhances this process by introducing a systematic quantification of risk through three key parameters: severity, occurrence, and detection.

By calculating the Risk Priority Number—the product of these three factors—potential hazards can be ranked according to their overall risk profile. The process of selecting critical points using our comprehensive hazard analysis was conducted. It involved identifying all potential CBRN hazards across the shopping center’s operations. The analysis reveals notable variations in vulnerability to the hazard, impact of the hazard, and likelihood of a hazard occurring across the assessed centers, however the main objectives of presented study has been achieved and the presented methodology introducing the innovative approach in places with food chains and can be used as a structured and practical approach in food safety assessment procedures, though further validation across a wider range of settings is required.

## 5. Conclusions

The findings of this study align closely with existing regulatory and standardization frameworks in food safety and defense. Within the European Union, food defense requirements are embedded in Regulation (EC) No. 178/2002 [27] and supported by the Commission’s guidance on mitigating risks of intentional adulteration, which emphasize vulnerability assessments and preventive controls. Similarly, the Codex Alimentarius ‘General Principles of Food Hygiene’ [28] now includes food defense considerations, underscoring the need for integrated approaches to both unintentional and intentional hazards. ISO 22000:2018 [26] also calls for a systematic risk-based approach to food safety management systems, with explicit reference to external threats and organizational resilience. By integrating HACCP, TACCP, and FMEA into a unified framework, this study operationalizes these regulatory expectations into a practical methodology. This not only facilitates compliance with EU and international requirements but also provides policymakers and industry stakeholders with a structured tool to harmonize food safety and food defense assessments in complex environments such as shopping malls.

## Figures and Tables

**Table 1:** Vulnerability assessment.

Scale	Vulnerability Assessment (V) Higher the Less We Are Protected Against This Hazard
1	Low (comprehensive security measures implemented and supervised)
2	Average (partial security measures implemented and supervised)
3	High (comprehensive unsupervised security)
4	Very high (unsupervised partial security)
5	Critical (no security system)

**Table 2:** Risk impact assessment.

Scale	The Impact of a Threat (W) Higher It Is the More Adverse Its Effects Are
1	Low (possibility of further functioning)
2	Average (difficulties in functioning)
3	High (excluding part of the facility from functioning)
4	Very high (over 50% of the facility is unfunctional)
5	Critical (complete exclusion from the functioning of the facility)

**Table 3:** Assessment of the likelihood of a hazard occurring.

Scale	The Likelihood of a Hazard Occurrence (PR) Higher It Is, As Potentially More Probable Threat May Occur
1	Low (potential number of events: no more than once a year)
2	Average (potential number of events: no more than one in 6 months)
3	High (potential number of events: no more than 1 per month)
4	Very high (potential number of events: no more than 1 per week)
5	Critical (potential number of events: 1 or more times a week)

**Table 4:** Critical control points assessed for risk calculation.

Symbol	Critical Control Point
A1	Vehicle access (monitoring, identification of unauthorized access, security) (Section 3.1.)
A2	Human access (Section 3.1.)
B	Food transportation inside the facility (Section 3.2.)
C	Security staff (Section 3.3.)
D	Back-office access (Section 3.4.)
E	Ready-made gastronomic products sold in an open manner (allowing free access to the product by third parties) (Section 3.5.)
F	Preparation of the consumption areas to serve guests eating meals on the spot (Section 3.6.)
G	Preparation of food products for distribution (Section 3.7.)
H	Ready-made products service (both eaten on site and take-away) (Section 3.8.)
I	The probability of inspection carried out by an individual posing as a Public Health Inspector (Section 3.9.)

**Table 5:** The average risk levels for particular critical control points.

Critical Control Point	The Average Vulnerability to the Hazard (*p*)	The Average Impact of the Hazard (W)	The Average Likelihood of a Hazard Occurring (PR)	Estimated Risk (R)
A1	2.67 ± 0.71	3.00 ± 1.23	2.78 ± 1.09	22.22
A2	2.89 ± 0.33	3.00 ± 0.50	3.11 ± 1.17	26.96
B	2.89 ± 0.93	2.67 ± 0.71	3.00 ± 1.12	23.11
C	2.11 ± 0.93	2.22 ± 0.44	2.00 ± 0.50	9.38
D	3.00 ± 0.71	2.89 ± 0.60	3.44 ± 1.01	29.85
E	2.11 ± 0.78	2.11 ± 0.78	2.33 ± 1.23	10.40
F	1.89 ± 0.33	1.67 ± 0.50	2.33 ± 0.71	7.35
G	2.00 ± 0.50	1.78 ± 0.67	1.89 ± 0.78	6.72
H	2.00 ± 0.71	1.89 ± 0.78	2.22 ± 1.20	8.40
I	2.89 ± 0.33	4.11 ± 1.45	2.22 ± 0.44	26.39

Values (mean ± standard deviation) were calculated based on the data from 9 diverse shopping centers in Poland, the Czech Republic, Spain, and Slovakia. Colors represent the risk level based on Figure 3.

## Data Availability

The original contributions presented in this study are included in the article. Further inquiries can be directed to the corresponding author.

## Abbreviations

The following abbreviations are used in this manuscript:

CBRN	Chemical, Biological, Radiological, And Nuclear (Threats)
CBRNe	Chemical, Biological, Radiological, Nuclear, And Explosives (Threats)
HACCP	Hazard Analysis and Critical Control Points
TACCP	Threat Assessment and Critical Control Points
FMEA	Failure Mode and Effects Analysis
EU	European Union
V	Vulnerability to Threat
W	Impact of a Threat
PR	Probabilistic Likelihood of Threat Occurrence
